# Optimising the treatment of chronic ischemic heart disease by training general practitioners to deliver very brief advice on physical activity (*OptiCor*): protocol of the systematic development and evaluation of a complex intervention

**DOI:** 10.1186/s12875-024-02655-3

**Published:** 2024-11-27

**Authors:** Sabrina Hoppe, Alicia Prinz, Rik Crutzen, Norbert Donner-Banzhoff, Andrea Icks, Daniel Kotz, Oliver Kuß, Ute Mons, Markus Vomhof, Stefan Wilm, Sabrina Kastaun

**Affiliations:** 1 Institute of General Practice (ifam), Patient-Physician-Communication Research Unit, Centre for Health and Society (chs), Medical Faculty and University Hospital Düsseldorf, Heinrich-Heine-University Düsseldorf, Düsseldorf, Germany; 2 Department of Health Promotion, Care and Public Health Research Institute (CAPHRI), Maastricht University, Maastricht, The Netherlands; 3 Institute of General Practice, Philipps-University Marburg, Marburg, Germany; 4 Institute for Health Services Research and Health Economics, Centre for Health and Society (chs), Medical Faculty and University Hospital Düsseldorf, Heinrich-Heine-University Düsseldorf, Düsseldorf, Germany; 5 Institute for Health Services Research and Health Economics, German Diabetes Center, Leibniz Center for Diabetes Research at Heinrich-Heine-University Düsseldorf, Düsseldorf, Germany; 6 Institute of General Practice (ifam), Addiction Research and Clinical Epidemiology Unit, Centre for Health and Society (chs), Medical Faculty and University Hospital Düsseldorf, Heinrich-Heine-University Düsseldorf, Düsseldorf, Germany; 7 Department of Family Medicine, Care and Public Health Research Institute (CAPHRI), Maastricht University, Maastricht, The Netherlands; 8 Institute for Biometrics and Epidemiology, German Diabetes Center, Leibniz Center for Diabetes Research at Heinrich-Heine-University Düsseldorf, Düsseldorf, Germany; 9 Centre for Health and Society (chs), Medical Faculty and University Hospital Düsseldorf, Heinrich-Heine-University Düsseldorf, Düsseldorf, Germany; 10 Department of Cardiology, Faculty of Medicine and University Hospital Cologne, University of Cologne, Cologne, Germany

**Keywords:** Chronic ischemic heart disease, Coronary heart disease, Physical activity, General practice, Primary care, Brief advice

## Abstract

**Background:**

Chronic ischemic heart disease (IHD) is one of the leading causes of morbidity and mortality. Physical activity (PA) is an effective secondary preventive strategy in IHD management. The German treatment guideline recommends that general practitioners (GPs) deliver PA advice to patients. This recommendation seems inadequately implemented, often due to GP’s insufficient specific training. International guidelines recommend training GPs in how to deliver such advice effectively and efficiently. Evidence is lacking on whether such training can enhance the frequency and quality of PA advice in routine care. The *OptiCor* project aims to develop and evaluate a GP training in the delivery of very brief PA advice to optimise the treatment of patients with IHD in general practice.

**Methods:**

*OptiCor* comprises three study phases according to the Medical Research Council recommendations for developing and evaluating complex interventions.

*Phase 1 (needs analysis):* A nationwide representative household survey will be conducted to collect data on the receipt of GP-delivered PA advice in people with IHD. Qualitative interviews and group discussions with GPs and people with IHD will help to explore, e.g., attitudes, experiences with, and barriers and facilitators of PA advice implementation or reception, respectively. Findings will inform the training development.

*Phase 2 (pilot):* A pragmatic cluster randomised controlled trial (cRCT) on the effectiveness of the developed training on proportions of GP-delivered PA advice during routine care of IHD patients will be piloted.

*Phase 3 (evaluation):* A full pragmatic cRCT will be conducted with patient-reported proportions of GP-delivered PA advice as primary endpoint. Collection of health economic and process-related data will facilitate a potential future broad implementation and health economic evaluation of the training.

**Discussion:**

If the developed training successfully improves proportions and quality of GP delivered PA advice to patients with IHD, it could serve as a low-threshold and sustainable strategy for implementing PA recommendations in the secondary prevention of IHD in routine GP practice.

**Trial registration:**

Work package (WP) 1, WP5, and WP6 have been prospectively registered at German Clinical Trials Register (WP1: DRKS00031304, 19/06/2023; WP5: DRKS00034641, 10/07/2024; WP6: DRKS00034642; 10/07/2024).

## Background

Chronic ischemic heart disease (IHD), also known as coronary heart disease, is one of the major causes of mortality and morbidity of patients worldwide [1]. In Germany, where this project takes place, IHD belongs to the most common diseases with a lifetime prevalence of approximately 9% in 40–79-year-olds [2]. IHD is associated with patients’ decreased quality of life [3] and an increased risk of co-existing mental health conditions such as depression [4, 5] and anxiety [6]. The annual economic burden of cardiovascular events on the German health care system amounts to €34.7 billion, equating to approximately 13% of Germany’s total health care expenditure [7].

Regular physical activity (PA) is an effective secondary preventive strategy in IHD management [8, 9] as it reduces the medium- to long-term risk of myocardial infarction, cardiovascular mortality, and all-cause hospitalisation [8]. Despite the beneficial effects of PA on IHD, approximately half of all people with IHD in Germany are not physically active [10]. In their Global Action Plan 2018–2030, the World Health Organization (WHO) emphasises the importance of increasing PA to prevent and treat IHD [11]. Correspondingly, the European Heart Network has asserted that routine assessment and advice on PA by health professionals should be a core element of cardiovascular patient care [9]. According to the current German guideline for the treatment of chronic IHD: “PA must be seen as an integral part of secondary prevention in people with IHD” [12].

IHD belongs to the most common treated conditions in general practice [13]. General practitioners (GPs) regularly engage with people with IHD (e.g., in Disease Management Programs (DMPs)). From the patients’ perspective, GPs are trusted sources of information on health behaviour [13]. Consequently, the WHO recommends the delivery of PA counselling as part of routine general practice, ideally as brief advice [11].

It has been shown that the offer of advice and support from a GP is effective at motivating sedentary patients to become more active [14–16], with a number needed to treat (NNT) of 12 to achieve long-term (12 months follow-up) behavioural change [14]. Brief advice is also a cost-effective way to improve PA among patients [17]. However, a recent systematic review encompassing studies with heterogeneous approaches of delivering advice (focusing on motivational interviewing, with different formats in terms of implementation, duration, and number of follow-up contacts) did not show clear effects of PA counselling on patients’ behaviour [18].

Studies that systematically focus on brief advice approaches (often also called brief interventions) as defined by the National Institute for Health and Care Excellence (NICE [19], definition see below), show that providing brief advice on PA can effectively enhance patients’ self-reported and objectively measured PA level in the medium term (up to six months) [20, 21]. However, taken together, the evidence so far does not allow clear conclusions about the long-term effects of brief advice [13]. Despite this, studies on other health behaviours, such as tobacco smoking and hazardous and harmful alcohol consumption, demonstrate that brief advice by GPs can positively impact patients’ health behaviour [22, 23].

In routine care, physician advice often consists of a non-specific discussion about the benefits of PA or risks of sedentary behaviour, which is often not done opportunistically [24]. Only a minority of patients receive elements of advice known to effectively increase the likelihood of behaviour change, such as concrete recommendations or referrals, assessment of PA level, and follow-up [24]. In addition, the majority of structured brief interventions tend to last 15 to 30 min and are thus still too lengthy for an implementation in routine GP practice [13].

As a result, it has been recommended to focus on very brief advice approaches, such as the “ask/assess, advise, assist” (3As; Ask/assess: ask for/assess the current PA level, advise: provide concrete advice to increase PA, assess: give concrete recommendation on opportunities to increase PA) structure, which takes only a few minutes [13]. According to the NICE guidelines, such interventions should be offered opportunistically, include an assessment of PA, involve advice on PA, along with negotiation or encouragement, with or without written support or follow-up [19]. Such approaches aim to avoid time-consuming negotiations about the patients’ motivation, and instead focus on providing concrete support tailored to the patients’ preferences, current level of activity, individual goals, and barriers. Such an approach might thus be more likely to be routinely implemented in GP practice and to reach the patients [13].

To date, evidence regarding the implementation of the clinical guideline recommendations on the delivery of PA advice in German general practice is sparse. The few existing studies have shown inconsistent findings on the prevalence of PA advice by GPs [25], and indicate that while people with chronic conditions such as IHD seem to be more likely to receive advice on PA, the overall implementation of such advice in German general practice appears to be insufficient [21, 26]. Furthermore, there is a lack of representative and current data on the structure and content of, and patients’ satisfaction with advice on PA provided by GPs to people with IHD, and on whether specific sociodemographic, socioeconomic or health-related person characteristics are associated with receiving PA advice. Such data could inform the development of tailored strategies to improve the implementation of PA advice in general practice.

Reported barriers preventing GPs from providing advice on PA include lack of knowledge and training on PA advice, time constraints, a lack of (information on) local services to refer patients to, and a lack of effective tools or information for patients [27]. The WHO und NICE guidelines recommend that physicians should receive training to effectively and efficiently provide advice on PA [11, 19]. According to the NICE guidelines, such a training should create an understanding of promoting PA as a preventive measure and responsibility in primary care, provide information on PA and recommendations for implementation, raise awareness of groups at risk of sedentary behaviour, and provide knowledge on target groups and their specific needs (e.g., people with disabilities), as well as on local sports services [19]. However, such training is either not included at all or only insufficiently included in the medical curriculum and in the continuing medical education of physicians in Germany.

In order for GPs to be motivated to participate in such specific training and for it to be implemented broadly, such training should aim to be as brief as possible, and aim to be carried out as a single-session training. However, there is a lack of evidence on whether such brief single-session training can effectively change GPs’ behaviour regarding the delivery of PA advice. To our knowledge, no randomised controlled trials on the effectiveness of such training on the proportions of GP-delivered PA advice have been conducted so far. A NICE review [19] suggests moderate evidence that GPs’ confidence and knowledge affects their ability to deliver PA advice, and that training may encourage GPs to deliver PA advice. However, none of the RCTs included in the review assessed the unique effectiveness of the provided training or its effects on the proportions of GP-delivered advice, and no study has been conducted in a routine care setting [19]. Additionally, most of the included studies provided little study details or did not apply a behaviour change theory to inform training development [28]. The use of the latter is a core element of behavioural studies as they require an appropriate method for characterising intervention elements and linking them to an analysis of the targeted behaviour [33]. Only one study focused on a specific patient population (obese patients), and none of the studies has been conducted in the German healthcare setting [19]. While studies related to other health behaviours (e.g., smoking) show that a brief training GPs can positively impact counselling behaviours [22], it remains unclear whether the results of these studies are transferable to PA.

### Objectives

The project *OptiCor* (“Optimising the treatment of chronic ischemic heart disease by training general practitioners to deliver very brief advice on physical activity”) [29] primarily aims at systematically developing a single-session, brief, tailored training for GPs in effectively and efficiently delivering advice on increasing PA to people with IHD, in line with the very brief 3As (ask/assess, advise, assist) method for structuring such advice, and using available theory and evidence [30]. The second aim is to evaluate the effectiveness of this training in increasing the proportions of GP-delivered brief PA advice (primary outcome of WP6) during routine consultations with people with IHD. Secondary outcomes include the proportions of GP-delivered advice including the assesment of patients' PA level, concrete recommendations or referrals concerning PA, and follow-up contacts to monitor or further discuss patients’ PA. The third aim is the collection of health economic, qualitative, and process evaluation data to facilitate a future implementation study including health economic evaluation.

## Methods/design

**Fig. 1:**
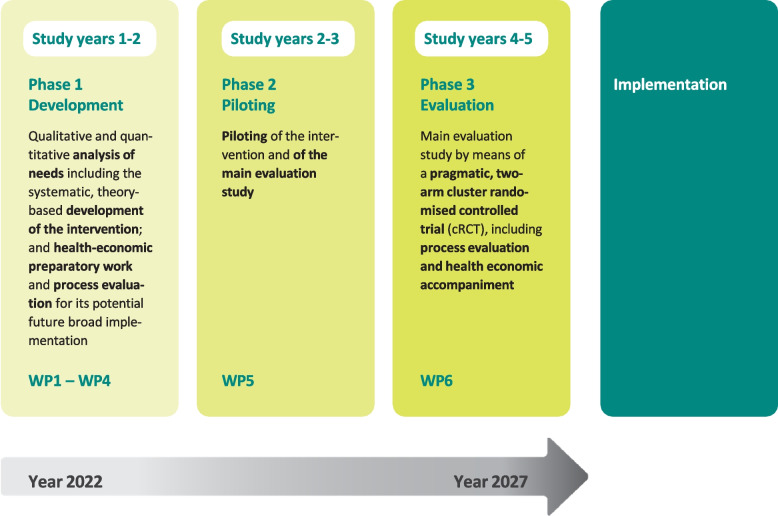
Key elements of the OptiCor project including the systematic development and evaluation of the complex intervention, based on the Medical Research Council (MRC) guidelines [31] (WP: work package)

The *OptiCor* project will comprise three study phases over a period of five years, and will follow the Medical Research Council (MRC) framework for the development and evaluation of complex interventions [31] (Fig. 1). An overview of all WPs (including expected duration, research questions/aims, and research methods) is presented in Table 1.

**Table 1:** Methodology of the work packages (WPs) 1–6 of the OptiCor project

**WP**	**Expected duration** *(incl. preparatory work and preleminary writing up of results)*	**Research questions/Aims**	**Research methods**
**Phase I: Quantitative and qualitative needs analysis including the systematic development of the intervention (training), and preparatory work for future health economic analyses** (study years 1–2)			
WP1: Cross-sectional population survey	24 months	*Primary research question*: • What is the proportion of people with IHD who report having received GP-delivered advice on PA according to the 3As method: ask/assess, advise, assist? *Secondary research questions*: • From the perspective of people with IHD, is there a wish for GP-delivered advice on PA, and if so, what specifically is the desired content of advice? • Are these outcomes associated with specific person characteristics (e.g., age, sex, education, body mass index, current PA level)?	• Nationwide, cross-sectional, computer-assisted, face-to-face household survey in approximately 1000 respondents aged ≥ 35 years with self-reported IHD and at least one GP visit since last remembered IHD-event
WP2: Qualitative study on GPs’ and patients’ attitudes, experiences, and needs	20 months	*Research aims* • What are the experiences of GPs and people with IHD with providing/receiving advice on PA? • Which attitudes, motivators, barriers and supporting factors for the routine implementation of PA advice by GPs can be identified? • What kind of needs do GPs haves with regard to a GP training on advising on PA?	• Individual problem-centred interviews with GPs (*n* = 8–12) and people with self-reported IHD (*n* = 8–12) • 4–6 focus group discussions with GPs (*n* = max. 10–12 per group) and people with IHD (*n* = max. 10–12 per group)
WP3: Development of the GP training	8 months	*Aims*: Development of a theory-based, brief, single-session, tailored GP training to deliver PA advice according the very brief 3As method during routine consultations with people with IHD	• The development will make use of the COM-B behaviour change theory [36], and will aim to describe the active elements of the intervention by using the behaviour change taxonomy [29] • The development will consider results from WP1 and WP2
WP4: Health economic preparatory work	18 months	*Aims (further aims of WP4 are listed under WP6)*: • Development of a DCE • Development of a questionnaire regarding health care utilisation in IHD patients	*DCE*: • Literature review • Qualitative semi-structured individual interviews with people with IHD based on an interview guide (*n* = 8–12) • Expert workshop (e.g., people with IHD, GPs, cardiologists) *Health care utilisation questionnaire*: • Literature review and consideration of previous work by members of the study team [51]
**Phase II: Piloting of the intervention and of the main evaluation study** (study years 2–3)			
WP 5: Pilot study	18 months	• What are the pilot implementation outcomes including the training of GP peer trainers, recruitment and randomisation processes, content and schedule of the training, materials, and methods of data collection among patients and GPs including pre-tests of study questionnaires? *Process evaluation*: • To what extent can the GP training content be transferred into practice? • What factors facilitate or hinder the transfer? • How do the GPs experience the implementation of brief PA advice? • Whatarethe patients' experiences with the GP advice they received (if so) and their met and unmet needs? *Health economic accompaniment*: • Is it feasible to elicit preferences on increased PA in IHD patients using the DCE?	• Pilot study as a “scale model” of one study cycle of the main pragmatic cRCT; One study cycle: 10 GP practices (intervention group *n* = 5, control group *n* = 5; pilot sample of 12 patients with IHD per practice = 120 patients) *Process evaluation*: • Process evaluation with semi-structured, problem-centred individual interviews with GPs (*n* = 6–8) and their patients with IHD (*n* = 6–8) Health economic accompaniment: • Pilot testing of the DCE (see WP4, *n* = 120 patients with IHD)
**Phase III: Main evaluation study by means of a pragmatic, two-arm cluster randomised controlled trial (cRCT), including process evaluation and health economic accompaniment** (study years 4–5)			
WP 6: Main evaluation study	24 months	*Primary research question*: • What is the proportion of people with IHD who report receiving brief advice on PA during a routine consultation with their GPs of the intervention group compared to GPs of the control group? *Secondary research questions*: • What is the proportion of people with IHD whose current PA level was assessed by the GP? • What is the proportion of people with IHD who received specific recommendations or information about duration, frequency, or type of PA exercise and/or patient information sheets with information about PA? • What is the proportion of people with IHD who received an offer for a follow-up appointment with the GP to monitor or discuss PA level? • How satisfied are the people with the advice they received (if so)? *Further research questions*: • What is the short-term training effect on GPs' reported attitude, opportunity, knowledge, and practical skills in providing advice on PA? *Process evaluation*: • What are facilitators and barriers of GPs to the routine implementation of PA advice, including reimbursement strategies for its routine provision? • What are the patients' experiences with the GP advice they received (if so; including facilitators and barriers for the implementation of the advice by their GPs)? *Accompanying health economic research*: • Which preferences do people with IHD have regarding outcomes on increased PA? • Is it adequate to assess health care utilisation in IHD patients with the developed questionnaire?	• Full pragmatic, two-arm cRCT with randomisation (1:1); data collection in 50 GP practices (25 per study arm) and in a study sample of *n* = 300 people with IHD per study arm *Process evaluation*: • Semi-structured qualitative interviews with people with IHD who received PA advice from a GP of the intervention group (*n* = 30–40) • Qualitative process evaluation with GPs (*n* = 8–12) *Health economic accompaniment*: • Evaluation of the DCE in people with IHD of the control group of the cRCT (see WP4, *n* = 300) • Piloting of the health care utilisation questionnaire in patients of the control group of the cRCT (*n* = 300)

*WP* Work package, *IHD* chronic ischemic heart disease, *PA* physical activity, *GP* general practitioner, *BCT* behaviour change taxonomy, *DCE* discrete choice experiment, *cRCT *cluster randomised controlled trial

## Phase I) Quantitative and qualitative needs analysis including the systematic development of the intervention (training), and preparatory work for future health economic analyses

### WP1: Cross-sectional population survey

To guide the development of the training intervention and to identify potential opportunities for improving the quantity and quality of PA advice, WP1 aims to assess national representative data on “how” GP advice is currently provided to people with IHD, and to explore specific individual person characteristics that might be associated with the receipt of and wish for such advice.

The primary outcome of WP1 is to determine the proportion of people in the population of Germany aged ≥ 35 years with self-reported IHD, who have had at least one GP consultation since the last remembered IHD-event (e.g., myocardial infarction, stent insertion), and who report having received GP-delivered advice on PA. This will be achieved by assessing the receipt of the 3As method (ask/assess, advise, assist). Secondary outcomes include exploring IHD-patients’ general wish for receiving GP advice on PA including desired content. Possible associations with sociodemographic, socioeconomic, and health-related individual characteristics (e.g., age, sex, body mass index (BMI), PA level) will be explored.

By means of computer-assisted personal interviews (CAPI) – conducted by a market research institute since June 2023 –, a sample of approximately 1,000 persons aged ≥ 35 years with self-reported IHD who had visited a GP since the last remembered IHD-event will be interviewed in a cross-sectional representative national household survey. Respondents will be selected by using a nationwide multistage, multi-stratified dual frame design: a composition of random stratified sampling (50% of the sample) and quota sampling (50% of the sample).

Based on a standard formula for cross-sectional surveys [32], the target size of 1,000 respondents with IHD will allow an estimation of the actual proportions of GP advice on PA with an absolute error of about 5%. This sample size also allows for some subgroup analyses with sufficient numbers of cases per subgroup.

The lifetime prevalence of IHD is about 9% in those aged ≥ 40 years in Germany [2]. The present study will include people aged ≥ 35 years. We also estimate a relatively conservative probability of 80% visiting a GP since the last remembered IHD-event. Older national survey data suggests that just over one third of people with IHD aged ≥ 65 years have received PA advice by a GP during the past year [26]. However, GP-delivered advice on other health behaviour occurs less often, and younger people might be even less likely to receive advice. The study therefore estimates a more conservative proportion of 20% of people with IHD who received GP-delivered PA advice including all elements of the 3As method (ask/assess, advise, assist). Based on this, about 14,000 individuals aged ≥ 35 years will be needed to identify the target sample. For this purpose, four established questions on IHD will be used which have been applied in several population health surveys before (e.g.,[2]), asking for a history of angina pectoris, myocardial infarction, bypass surgery or coronary stent insertion. A modified version of the validated single-item question of Milton et al. will be used to record the current PA level [33]. The CAPI questionnaire on primary and secondary outcomes was developed in a multi-professional team (e.g., GPs, psychologists, epidemiologists, public health researchers) and pre-tested in patients with IHD (the full questionnaire translated into english has been published at Open Science Framework (OSF) [34]).

### Analyses WP1

A detailed study and analysis protocol will be published at OSF prior to the statistical analyses. Descriptive statistics will be used to analyse prevalence data on primary and secondary outcomes. Non-adjusted and adjusted multinomial regression analyses will be used to explore associations between respondents’ characteristics (e.g., age, sex, BMI) and receipt of GP advice as well as with the expressed wish for receiving such advice. Face-to-face data sampling usually produces few missing data. If missing data occur to a relevant extent, non-response analyses will be conducted and the application of multiple imputation methods will be considered.

### Current status

At the time of submission of this protocol, data collection has already been completed. The analyses are still being conducted.

### WP2: Qualitative study on GPs’ and patients’ attitudes, experiences, and needs

In WP2, attitudes, motivation and experiences of GPs with the delivery of PA advice to their people with IHD will be explored to understand barriers and facilitators to routinely delivering such advice. In addition, we aim to identify GPs’ needs concerning the concept, content, and conditions for an optimal brief training on the delivery of PA advice. Complementarity, attitudes, acceptability, needs, and barriers of people with IHD towards the receipt of PA advice in the general practice setting will also be assessed, what corresponds to the contents of the COM-B Model ( [35], definition see below). For this purpose, individual problem-centred interviews [36] and focus group discussions [37, 38] with GPs and people with IHD, respectively, will be conducted based on interview and focus group guides. These guides were developed in a multi-professional study team, including patient representatives and GPs, and have been pre-tested.

In order to achieve a diverse range of professional experience, sociodemographic characteristics, general practice characteristics, and interest in PA among GPs, and of sociodemographic, socioeconomic, and health-related characteristics, as well as interest in PA among people with IHD, these groups will be selected using a purposive sampling strategy [39]. For maximum structural variation, case contrast strategies are provided, which also serves to assure the quality [40]. The process of sampling is supported by the use of a short questionnaire. This questionnaire and the interview and focus group guides have been published at OSF [41].

A number of four to six focus groups each with GPs and patients, respectively, with a maximum of ten to twelve participants per group, and eight to twelve individual interviews each with GPs and patients are expected to be sufficient. Data collection will continue until reaching data saturation [42]. GPs will be recruited through addressing regional quality circles of GPs (association of GPs who meet regularly to share and reflect on their everyday practice [43]), representatives of practice and GP networks, the teaching and research practice network of the study institute, and from the NRW General Practice Research Network [44]. Patients will be contacted via self-help or rehabilitation groups in the Rhine-Ruhr region. In addition, study information in health care facilities and public institutions will be used to reach people who are preferably not members of self-help groups or cardiac sports groups. Experienced moderators from the study institute will conduct the focus group discussions. Data will be collected in an iterative cyclical process. Saturation marks the point in the iterative process of data collection and evaluation at which additional data collection does not lead to any further gain in knowledge according to the research question. The interviews and group discussions will be audio-recorded and transcribed verbatim, according to simple scientific transcription rules [45]. Postscripts document the atmosphere, the conversation process, interactions, specifics, and disruptions.

### Analyses WP2

The content-analytical data evaluation of the audio-recorded and verbatim transcribed pseudonymised data will be carried out using the software MAXQDA [46] in a content-structuring procedure [47] by a multi-professional study team (e.g., GPs, patient representatives, psychology, social science, public health). Content-structuring qualitative content analysis is, in addition to evaluative and type-forming qualitative content analysis, a procedure of category-based methods for the systematic analysis of qualitative data [47]. These evaluation procedures on category-based methodology is a language-related, rule-guided systematic scientific method with the aim of pragmatic reduction of complexity [47].

### Current status

At the time of submission of this protocol, the data collection has been completed. The analyses of GP data have also been completed, analyses of patient data are still being conducted.

### WP3: Systematic development of the GP training

WP3 focuses on the synthesis of results from WP1 and WP2 to inform the systematic development of a brief, single-session, tailored GP training on the delivery of PA advice to people with IHD according to the 3As method.

In a strategy workshop, results of WP1 and WP2 will be compared and connected, using visual representations (e.g., acyclic behaviour change diagrams [48]), discourse and expert consensus techniques.

The interdisciplinary study team, comprising experts in research on behaviour change, health services and public health, will develop a training manual in collaboration with GPs and patient representatives, with provisions for future adjustments for sustainability, such as online booster modules and good practice videos. The COM-B (capability, opportunity, and motivation-behaviour) model [35] will serve as theoretical framework for the development of the training, and the behaviour change taxonomy (BCT) will be used to describe active elements of the training [28]. According to COM-B, the interplay between capability (C), opportunity (O), and motivation (M) influence behaviour (B), and a specific behaviour in turn influences these factors [35]. The training will be developed to address “capability and motivation” by providing knowledge and practical skills to deliver PA advice. By using the 3As (ask/assess, advise, assist) method as a very brief, less time-consuming method of delivering advice, which can be more easily integrated into daily practice, the training will also aim to influence “opportunity”. The definition and description of potentially active training elements according to the BCT [28] will also be part of WP3. Training materials and case vignettes for simulated role-plays using simulation patients will be developed in collaboration with the “communication in medical education” team of the Heinrich-Heine University. Experienced GPs and peer-trainers from the study institute will review the training manual and didactic methods.

### Current status

At the time of submission of this protocol, a first draft of a training manual has been developed as described above.

### WP4: Accompanying health economic research

WP4 aims at developing a (web-based) discrete choice experiment (DCE) which will identify preferences regarding IHD patients’ outcomes on increased PA, and to develop a questionnaire that accurately assesses health care utilisation in people with IHD. Findings of WP4 will thus help to inform future RCTs by providing the most preferred patient-relevant outcomes for PA based interventions and to inform future health economic evaluations. The development of the DCE will adhere to recent recommendations [49].

First, a literature review and eight to twelve qualitative semi-structured individual interviews with people with IHD based on an interview guide will be conducted to identify relevant outcomes on increased PA. Second, these identified outcomes will be discussed with experts (e.g., people with IHD, GPs, cardiologists) in a joint workshop. In addition, the findings on relevant patient outcomes will be considered in the process of training refinement described in WP5. The experts will select the most important outcomes that will be used as attributes in the DCE. Alongside, a health care utilisation questionnaire will be developed based on a literature review and previous work by members of the study team [50].

The DCE will be pilot tested in people with IHD of the pilot study (see WP5, *N* = 120), and evaluated in patients of the control group of the main cluster randomised controlled trial (cRCT) (WP6, *N* = 300). The health care utilisation questionnaire will be pilot tested in patients of the control group of the main cRCT. IHD patients will receive a printed version of the questionnaires as well as a link enabling them to fill in a web-based version, alternatively. This will help to estimate the acceptance of different modes of assessment and to choose the optimal mode of assessment for the evaluation of the DCE which will follow in WP6 (*n* = 300). To avoid influences on the main study outcomes of the cRCT in WP6, patients will be asked to complete the DCE and health care utilisation questionnaires at home, following the collection of the primary and secondary outcomes of the cRCT.

### Analyses WP4

Transcribed individual interviews will be analysed by qualitative content analysis. Outcomes will be identified according to the suggested criteria for patient-relevant outcomes by Nano et al. [51]. Preference weights of the DCE will be derived by a conditional logit regression model and by latent class analysis considering preference heterogeneity. Health care utilisation data will be analysed descriptively by means, standard deviations, and distributions of quantities.

### Current status

At the time of submission of this protocol, WP 4 is still ongoing.

## Phase II) Piloting of the intervention study and of the main evaluation study

### WP5: Pilot study

Based on preliminary work of the study team [22] and as recommended according to the MRC framework [31], a feasibility study testing the practicality of the planned evaluation study (WP6) is not considered necessary. Instead, a pilot study will be carried out to evaluate implementation outcomes, including the training of GP peer trainers, recruitment and randomisation processes, content and schedule of the training, materials, methods of data collection among patients and GPs including pre-tests of the study questionnaire and potential need for sample size adjustment. The pilot study will essentially function as a “scale model” of one study cycle of the planned pragmatic, two-arm, clustered randomised controlled trial (cRCT). For each study cycle, ten GP practices will be randomised (1:1) into either the intervention or control group. A study cycle is defined as a period of 5 weeks of face-to-face, practice-based data collection in people with IHD immediately following consultation with GPs who have been trained (intervention group, *n* = 5 practices) and a period of 5 weeks of face-to-face data collection in people with IHD immediately after consultation with GPs who have not been trained (control group, n = 5 practices). Per practice, approximately twelve people with IHD will be recruited, resulting in a pilot sample of 120 people with IHD.

A process evaluation with semi-structured, problem-centred individual interviews with GPs of the pilot study and with their patients with IHD who received PA advice will also be conducted. The aim is to understand: A) to what extent training contents could be implemented into practice, which factors facilitated or hampered the transfer, and how GPs experienced the delivery of very brief PA advice, and B) patients’ experiences with received advice, and their met and unmet needs. Experiences with the study procedures will be explored in both groups. Approximately six to eight interviews per group are expected to be sufficient. Data collection will continue until reaching data saturation [42] (for methodological details see WP2). Based on the insights gained from these interviews, study processes and the training manual will be further refined.

### Analyses WP5

The process evaluation data will be analysed as described in WP2.

### Current status

At the time of submission of this protocol, WP 5 is still ongoing and will be finished by the end of 2024.

## Phase III) Main evaluation study by means of a pragmatic, two-arm cluster randomised controlled trial (cRCT), including process evaluation and accompanying health economic research

### WP6: Main evaluation study

Following successful implementation of WP1 to WP5, WP6 aims to evaluate the effectiveness of the developed brief, single-session, tailored GP training in increasing patient-reported proportions of GP-delivered brief advice on PA to people with IHD.

The primary outcome will be the (1) proportion of people with IHD who report having received PA advice during a routine consultation with their GP. Secondary outcomes will include the proportion of patients reporting (2a) the assessment of their PA level by the GP, (2b) the receipt of a concrete recommendation on PA (e.g., the receipt of concrete information on duration, frequency or type of PA exercise and/or patient information leaflets with information on PA), (2c) receipt of an offer for a follow-up appointment with the GP to monitor or discuss the patients’ PA level, and (2d) in the subgroup of patients with IHD who reported that they had a conversation with their GP on PA: satisfaction with the advice received. Another outcome will be (3) the short-term effect of the training on GP-reported attitude towards, opportunity and knowledge of, as well as practical skills in the delivery of PA advice, according the COM-B theory [35].

For these purposes, a pragmatic, two-armed cRCT with 1:1 randomisation will be conducted in GP practices. Since details of WP6 may change after WP5 has been completed, the design of WP6 will only be described briefly.

**Fig. 2:**
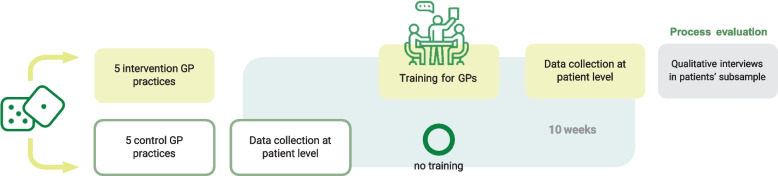
Study flow of the planned cluster randomised controlled trial (O = no training). A study cycle consists of 10 weeks of data collection: 5 weeks patients of “control” and 5 weeks in patients of “intervention” general practitioners (GPs). In total there will be five cycles resulting in 50 practices (25 intervention and 25 control practices)

Primary and secondary outcomes will be collected over a five-week period of face-to-face, practice-based data collection in people with IHD immediately following their consultation with GPs who have received the developed training on PA advice (intervention group). Data collection in people with IHD immediately after their consultation with untrained GPs (control group) will also conducted in a five-week period, resulting in a total period of 10 weeks of data collection for one full study cycle (Fig. 2).

As described in WP1, we calculate with an estimate of 20% of patients who report the receipt of brief PA advice during their GP visit without the GP having been trained. Another study showed that a brief single-session GP training can increase patient-reported rates of GP-delivered stop-smoking advice by 20%-points on average [52]. Training GPs to deliver PA advice might be more challenging, e.g., because PA recommendations are more complex than simple advice on smoking cessation methods. Training on PA advice is thus declared to have a clinically relevant effect if higher rates of delivered PA advice of at least 13%-points can be achieved in the intervention group (33%) as compared to the control group (20%), corresponding to an odds ratio of 1.97. Sample size calculation is based on the standard χ2-test in a four-fold-table and a multiplicative correction (= 1 + 0.05*(12–1) = 1.55, i.e., assuming an intraclass correlation coefficient of 0.05 and twelve IHD patients/practice) by the design effect to account for clustering. Including a total of 600 people with IHD in 50 practices (25 per study arm) will yield a power of at least 85% in the primary analysis of the intervention effect in a logistic regression model with a random intercept effect accounting for the nested design, while adjusting for patient and practice characteristics.

It is expected that around twelve people with IHD can be recruited within a period of five weeks, hence a total of five study cycles (with a total of 25 control practices and 25 intervention practices) will be needed to reach the target sample of 600 people with IHD. Periods of data collection in both groups will be temporally close to each other to avoid influences by fluctuations in patient flow, but cannot be conducted in parallel due to logistical reasons. The potential bias which may arise from the presence of a researcher collecting data in the practices is assumed to be equally distributed over the study arms. Patients will be masked to the purpose of the study until the end of the data collection. Due to the pragmatic nature of the study, neither GPs nor researchers can be fully masked to the allocation of study arms. To mitigate the risk of contamination, standard operation data collection procedures will be developed, and researchers will not be involved in the delivery of the training.

The control group will not receive a "placebo" intervention as this may lead to lower participation rates and pose a risk of bias (e.g., if a training on the delivery of stop-smoking advice would be offered to controls, GPs might be sensitised to provide advice on other detrimental health behaviours). However, once data collection in their patients is completed, GPs of the control group will immediately be offered the same training as the intervention group. This approach will ensure high retention rates in the control group.

GPs who have not participated in a program to promote PA within the last 5 years will be eligible. There are no other inclusion or exclusion criteria for GPs.

Patients will be enrolled in the study if they are ≥ 35 years old and if they have a clinical diagnosis of IHD according to International Classification of Diseases-10 I25.-I25.9. Due to data protection regulations, it will not be possible to learn about the presence of a clinical IHD diagnosis until the patient has provided written informed consent to participate. All consecutive patients willing to participate will therefore be screened during the study interview for a self-reported IHD diagnosis made by a physician, comparable to standards in health surveys [e.g., [2]]. Only participants screened positive for IHD will be interviewed on the study outcomes. All participants will be asked for their permission to verify their IHD screening result with the medical record obtained from the practice nurse. Only patients with a clinical diagnosis will be included in the final analyses. If the pilot study shows that there is a high level of correlation between self-reported IHD and validated IHD, strategies will be developed to include people who only report IHD in their self-report in the statistical analysis of the evaluation study (e.g., sensitivity analyses). The inability to provide informed consent (e.g., language barriers) and physical handicap impeding PA will be exclusion criteria for patients. GP practices will be recruited from the online register of the regional Association of Statutory Health Insurance Physicians NRW, and from the NRW General Practice Research Network [44].

With regard to a potential broader implementation and dissemination of the training, a qualitative process evaluation (comparable to WP5) with approximately eight to twelve GPs will accompany the cRCT to explore facilitators and barriers of GPs to the routine implementation of PA advice, including reimbursement strategies for its routine provision. In addition, semi-structured qualitative telephone interviews will be conducted in a subsample of 30 to 40 people with IHD who received PA advice from a GP of the intervention group on their motivation to become more active, and on barriers and facilitators to implement the recommendations of their GP into their daily lives, one month following the GP consultation (for methodological details see WP2 and WP5).

### Analyses WP6

A detailed study and analysis protocol will be published at OSF prior to the statistical analyses. The data analysis will follow the hierarchical clustered structure of the study (patients nested within practices). Logistic mixed-effects regression models will be applied to analyse the primary outcome, with a fixed effect for the group (intervention versus control) and a random intercept for the practices. The same model will be used on the secondary outcomes. Models will be adjusted for pre-defined potential confounders: patients’ age, sex, education, level of PA, and BMI. All patients will be included in an intention-to-treat analysis. Based on experiences from another cRCT [52], missing data on the primary outcome is assumed to be minimal as data will be collected through face-to-face interviews. However, multiple imputation will be applied to impute missing data if needed.

### Current status

At the time of submission of this protocol, WP 6 hat not started yet.

## Discussion

PA has been demonstrated to have substantial secondary preventive effects in IHD [8, 9]. Despite clinical guidelines advocating for the delivery of brief advice on PA in the primary care of people with IHD [12], this recommendation seems to be insufficiently implemented [26], often due to a lack of training [27]. While international guidelines recommend training GPs to provide such advice [11, 19], evidence is still lacking on whether brief training can effectively improve the proportions and quality of GP-delivered PA advice in routine healthcare. The *OptiCor* project intends to bridge this gap. This study protocol describes the systematic development and evaluation of a complex intervention for optimising the treatment of people with IHD by training GPs to effectively and efficiently deliver very brief advice on PA.

A strength of the study is the use of the MRC framework [31] and its core elements to systematically develop and evaluate the intervention. The development follows the COM-B behavioural model [35], and the BCT [28] will be used to describe the active elements of the training. This will make the intervention replicable in its design and results. In addition, the systematic inclusion of GPs and people with IHD (including patient representatives) in the study design and development of the training, as well as the pragmatic evaluation approach with relatively unselected patients and under real-world practice conditions, will strengthen the external validity of the results and may provide methodological and procedural data for future implementation studies.

The study also has limitations. Firstly, no medium- or long-term follow-up of patient or GP behaviour is planned for the cRCT for practical reasons. Thus, only short-term effects of the intervention on the GPs’ behaviour can be determined. Nevertheless, the study shows whether training for GPs has the potential to increase the proportions of GP-delivered PA advice in general. If this proves to be true, strategies can be devised (e.g., blended-learning refresher modules) to ensure sustainable training effects. Secondly, only GPs and people with IHD from one federal state (NRW) will be included in the study, limiting the study’s generalisability across Germany. However, NRW is the most populous German federal state, with a broad socioeconomic variability, and the recruitment will be conducted across urban and rural areas to ensure diversity. Thirdly, a selection bias could occur if particularly engaged or interested GPs and people with IHD are more likely to participate in the respective studies. In WP1, non-response analyses will be conducted and the application of multiple imputation methods will be considered. In WP2, one strategy to mitigate potential selection bias is to recruit complete GP quality circles for the focus groups. GPs in these quality circles are assumed to have different positions and motivations with regard to PA advice. In WP5 and WP6, randomisation should ensure equal distribution of such attitudes over both study arms. Nevertheless, a potential selection bias of GPs with a particular interest in lifestyle counselling or PA cannot be completely avoided. We will further explore and apply strategies to minimise selection bias by recruiting less motivated GPs (e.g., by “cold calling”). Lastly, as no physiological IHD markers or PA levels of patients will be investigated, effects of GPs' advice on the PA levels of people with IHD or on IHD progression will remain unclear for this study. Nevertheless, as reported in the background of this protocol, evidence is strong that GP advice increases PA levels at least in the medium term, and that regular PA improves the clinical outcomes of IHD [14–16].

## Conclusion

If the systematically developed training increases the frequency and quality of GP-delivered PA advice for secondary prevention of IHD, it could provide a low-threshold and sustainable strategy for implementing PA guideline recommendations for the management of IHD in general practice. The short duration of the training could further facilitate widespread implementation in the continuing education of GPs. It would also be conceivable to transfer the training on the delivery of very brief advice on PA to disease management of other chronic conditions (e.g., arterial hypertension, type 2 diabetes mellitus, depression), as well as to other healthcare settings (e.g., hospital, other specialists).

## Data Availability

The data which will be collected with this study are third-party data and will be available to researchers on reasonable request by contacting the principal investigator (sabrina.kastaun@med.uni-duesseldorf.de). All proposals requesting data access will need to specify how it is planned to use the data, and all proposals will need approval of the study team before data release.

## Abbreviations

IHD: Chronic ischemic heart disease
PA: Physical activity
GP: General practitioner
cRCT: Cluster randomised controlled trial
WP: Work package
DRKS: German Clinical Trials Register
WHO: World Health Organization
DMP: Disease Management Program
NNT: Number needed to treat
NICE: National Institute for Care Excellence
3As: Ask/Assess, advice, assist
MRC framework: Medical research council framework
BMI: Body mass index
CAPI: Computer-assisted personal interview
OSF: Open Science Framework
NRW: North Rhine Westphalia
BCT: Behaviour change taxonomy
DCE: Discrete choice experiment
